# The Oxidation of ZrB_2_/MoSi_2_ Ceramics in Dissociated Air: The Influence of the Elaboration Technique

**DOI:** 10.3390/ma17153818

**Published:** 2024-08-02

**Authors:** Ludovic Charpentier, Pedro Miranda, Hugo Tallaron, Florencia M. Nogales, Álvaro Sández-Gómez, Eric Bêche, Marianne Balat-Pichelin

**Affiliations:** 1 PROMES-CNRS, 7, Rue Du Four Solaire, 66120 Font-Romeu Odeillo, France; hugo.tallaron@promes.cnrs.fr (H.T.); eric.beche@promes.cnrs.fr (E.B.); marianne.balat@promes.cnrs.fr (M.B.-P.); 2 Department of Mechanical, Energy and Materials Engineering, Industrial Engineering School, University of Extremadura, Avda. de Elvas s/n, 06006 Badajoz, Spain; pmiranda@unex.es (P.M.); florenciamn@unex.es (F.M.N.); alvarosg@unex.es (Á.S.-G.)

**Keywords:** oxidation, additive manufacturing, dissociated atmosphere, scanning electron microscopy

## Abstract

In order to investigate the most extreme conditions in which materials potentially applicable in reusable thermal shields can be operated, ultra-high-temperature ZrB_2_ ceramics with 20 vol.% MoSi_2_ were prepared using two different techniques, cold isostatic pressing (CIP) and robocasting (RC, an additive manufacturing technique), followed by consolidation using pressureless spark plasma sintering (SPS). The oxidation behavior of the resulting materials was analyzed in low-pressure dissociated air at three different temperatures, namely 1800, 2000 and 2200 K. Using XRD and surface and cross-section SEM (coupled with EDS), zirconia was found to form at all three temperatures, while silica was only present at 1800 K, with gaseous SiO forming at a higher temperature. The elaboration technique influences the density of the ceramic, and less dense materials undergo deeper oxidation. This investigation suggests that 2000 K is already beyond the maximum temperature threshold at which damage to ceramics is limited by the formation of protective silica. This study confirms that the selected material is a promising candidate for thermal protection applications.

## 1. Introduction

High-temperature thermal protection systems (TPSs) are critical in many industrial applications, such as aerospace and energy generation. They typically rely on passive thermal insulation, which is limited by the oxidation and ablation resistance of the constituent materials. The AM-ACTS (Additive Manufacturing—Actively Cooled Thermal Shield) M-era.Net project [1] is developing novel high-performance thermal shields that can be actively cooled by circulating an appropriate fluid through a bioinspired internal microchannel network, with additive fabrication (3D printing) of the TPS elements out of ultra-high temperature ceramics and refractory metals. This will enable the sustainable and environmentally friendly production of atmospheric re-entry shields for reusable space crafts, turbine blades, rocket engines, reactor walls and solar receivers with an improved maximum service temperature and/or service lifetime at that temperature, boosting energy efficiency and reducing maintenance costs in these industrial applications, among others.

The specifications for active cooling mainly depend on the maximal operating temperature that the thermal shield materials can support without their reusability being compromised by damage. The experimental work presented here aims to identify the operating conditions for one of the materials envisaged, a composite ceramic of zirconium diboride ZrB_2_ incorporating 20 vol.% of MoSi_2_ (these samples will be labeled as ZBM from hereon). This material will be elaborated upon using two methods—(conventional) cold isostatic pressing (CIP) and (additive manufacturing) robocasting (RC), also known as DIW (direct ink writing). ZrB_2_ was chosen due to its high melting point (>3270 K [2]) and low theoretical density (6.09 g cm^−3^ [3]), making this ultra-high-temperature ceramic (UHTC) interesting for aerospace applications. Nevertheless, pure ZrB_2_ is hard to densify and forms porous zirconia ZrO_2_ and volatile boron trioxide B_2_O_3_ in oxidizing atmospheres, leaving this UHTC unprotected from severe oxidation in air at high temperatures [2]. Incorporating silicon additives such as MoSi_2_ [2] or SiC [3,4,5] favors densification and can decrease the oxidation rate due to the formation of a protective silica SiO_2_ layer at up to 1800 K (passive oxidation) in air at atmospheric pressure [4]. Nevertheless, at higher temperatures and/or lower oxygen pressures, gaseous SiO may form instead of SiO_2_, and more significant degradation is observed as a result of this so-called active oxidation [3,4,5].

Most investigations of the oxidation behavior of UHTC materials have been performed in non-dissociated air at atmospheric pressure (as in [2,3]). Nevertheless, during spatial re-entry, thermal shields are exposed to air at low pressure, with oxygen molecules dissociating into atomic oxygen due to the shockwaves generated by the fast velocity of the spaceship [6]. Therefore, here, we investigated the oxidation behavior of ZBM samples in a dissociated atmosphere, comparing RC samples to CIP ones. As further explained, the different densities obtained using these different techniques will impact the oxidation paths inside the materials.

## 2. Materials and Methods

High-purity commercial powders of ZrB_2_ grade B (>99%, d50 ~1.5–3 µm, ρ = 6.10 g cm^−3^) and MoSi_2_ grade B (>99%, d50 ~3.5–4 µm, ρ = 5.9 g cm^−3^) (Höganäs AB, Höganäs, Sweden) were used to prepare the ZrB_2_ + 20 vol.% MoSi_2_ mixtures. These were ball-milled in ethanol for 24 h and subsequently dried on a hot plate with continuous magnetic stirring. Conventional pellets were prepared through a process of uniaxial pressing (15 MPa), followed by cold isostatic pressing at 350 MPa.

The robocast samples were prepared based on water-based suspensions of the same powder mixture with a 40 vol.% solid loading. The aqueous medium was a pre-mixed solution of 25 wt% Pluronic F-127 (Sigma-Aldrich, Merck Group, Darmstadt, Germany) flocculant in distilled water. The solid loading was added in 3 steps, and the mixture was centrifuged after each addition for 1 min at 2000 rpm using a centrifugal planetary mixer (ARE-250: Thinky Corp, Tokyo, Japan). The resulting inks were extruded through a 0.41 mm diameter conical nozzle according to a typical RC/DIW process using a commercial 3D printer (Delta Wasp 2040: WASP, Massa Lombarda, Italy) and an in-house-adapted extruder and deposited layer-wise into the desired shape. Ultimaker Cura (version 5.4.0) was used as the printing software, with the following main printing parameters set: layer height, 0.2 mm (0.3 mm for the first layer); deposition speed, 5 mm s^−1^; travel speed, 10 mm; retraction speed, 12.5 mm s^−1^; retraction distance, 0.5 mm; infill overlap, 15%; and infill flow, 110%. Printing was carried out inside a paraffin bath to prevent the sample from drying unevenly, which can lead to the appearance of fissures. After deposition, the specimens were slowly dried for 24 h in a relatively humid atmosphere and then de-bound in a retort furnace under a vacuum for 2 h at 180 °C and for 2 h at 420 °C (with ramps of 1 °C min^−1^) to remove residual water and pyrolyze the Pluronic, respectively.

Both the RC- and CIP-formed samples were sintered using a spark plasma sintering (SPS) furnace (HP-D-10: FCT Systeme GmbH, Frankenblick, Germany) without applying any pressure to the samples, thanks to an appropriately prepared graphite die. They were heat-treated in a dynamic vacuum atmosphere (i.e., 6 Pa) at 1900 °C for 15 min after a plateau at 1500 °C for half an hour to safely remove the boria without cracking the specimens.

Oxidation inside a dissociated atmosphere was performed in the experimental facility MESOX (Moyen d’Essai Solaire d’OXydation—Oxidation Solar Test Facility), conceived in the PROMES-CNRS laboratory, with solar concentration used to heat the ceramic samples and a microwave guide used to generate atomic oxygen plasma in the reduced-pressure air (from 100 to 5000 Pa) [6,7,8,9]. ZBM was oxidized at a total atmospheric pressure of 1000 Pa and an airflow of 4 l h^−1^. The microwave generator was used with an output power of 300 W, enabling the dissociation of 70% of the oxygen molecules [6]. Three temperatures for passive to active oxidation were investigated: 1800, 2000 and 2200 K. For each temperature, a single different ZBM sample was held for 5 min at the requested plateau. The samples were weighed before and after the oxidation tests, and the variation in the mass of their oxidized surfaces was reported in mg cm^−2^. The accuracies of the temperature and weight measurements were ±20 K and ±0.1 mg, respectively.

X-ray diffraction (XRD) was performed using a PANalytical (Malvern Panalytical Ltd., Malvern, UK) X′Pert Pro diffractometer (Cu-Kα radiation with λ = 0.15418 nm). The setup was θ-θ-symmetric, and the scans were performed over a 2θ range from 10° to 90°. The step size and the time per step were fixed at 0.017° and 50 s, respectively. Scanning electron microscopy coupled with energy-dispersive X-ray spectroscopy (SEM/EDS) was carried out using the JEOL IT800 SHL LV equipment (JEOL Ltd., Tokyo, Japan) on the surfaces and cross-sections of the oxidized samples. Cross-section images of the samples were taken, which were cut along their diameter, rubbed in resin and polished to a mirror-like finish (with the polishing completed using a 1 µm diameter diamond suspension). The micro-Raman experiments were performed using a HORIBA LabRAM HR Evolution Raman spectrometer (HORIBA Ltd., Kyoto, Japan) equipped with an optical microscope (Olympus K.K. BX 41), a charge-coupled device detector (1024 × 256 pixels, 26 × 26 µm^2^ by pixel) and two laser beams. The Raman shifts were calibrated using a Si standard sample. The measurements were performed at room temperature. The Raman spectra were recorded in the wavenumber range (spectral region) from 80 to 1100 cm^−1^. The excitation source was a 633 nm laser beam, and the laser power was reduced to about 5 mW (with 25% filter strength) to avoid adverse laser-induced effects (e.g., surface heating). No temperature effects (such as a shifting of the peaks or differences in the intensity ratios compared with the reference spectra) were observed. The Raman spectra were collected at the focal point of a ×50 objective lens (with a numerical aperture (NA) of 0.75) and with a spectral resolution close to 1 cm^−1^/pixel. A 600 g/mm grating and a confocal pinhole value of 100 µm were selected. The acquisition time and the number of acquisitions per spectra were fixed at 10 s and 2, respectively. The Raman spectra were collected using the HORIBA LabSpec 6.0 software. The spectra background was removed.

## 3. Results

### 3.1. Observations after the Oxidation Tests

**Figure 1:**
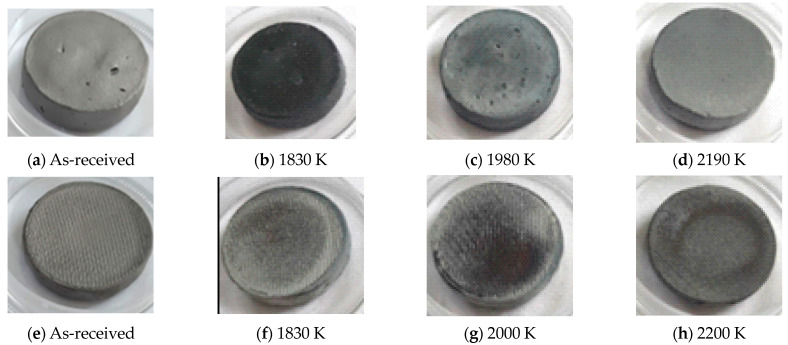
Pictures of the ZBM samples, CIP (**a**–**d**) and RC (**e**–**h**), as-received (**a**,**e**) and after oxidation in dissociated air at P = 1000 Pa at 1830 (**b**,**f**), 1980–2000 (**c**,**g**) and 2190–2200 K (**d**,**h**).

**Figure 2:**
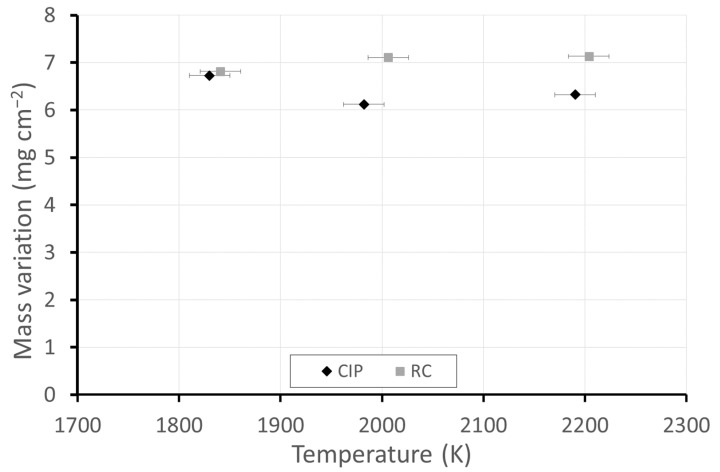
Mass variations of the ZBM samples, CIP and RC, after oxidation in dissociated air (P = 1000 Pa) at various temperatures.

Figure 1 presents pictures of the CIP and RC ZBM samples, as-received and after oxidation in dissociated air at three different temperatures, with the measured temperature denoted. Figure 2 presents the evolution of the mass variations according to temperature. Two different behaviors are observed: the CIP samples have darker surfaces after oxidation at 1830 K and clearer surfaces with a lower mass gain at 1980 K and 2190 K than at 1830 K, whereas the RC samples are not darkened at 1830 K and demonstrate a higher gain in mass than the CIP samples that uniformly increases with the testing temperature.

### 3.2. Characterization—CIP Samples

**Figure 3:**
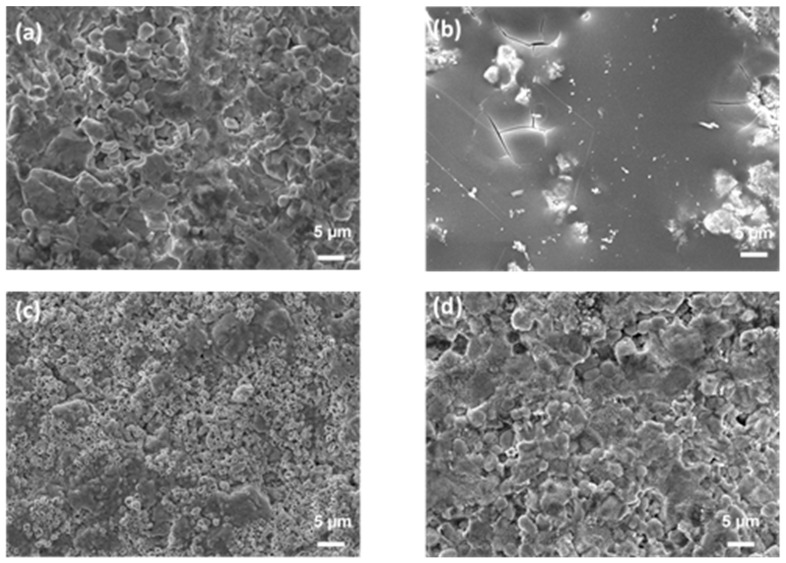
SEM images (secondary electron mode, acc. voltage: 15 kV, mag. ×2000) of the surface of CIP ZBM, (**a**) as-received and after 5 min of oxidation in dissociated air (P = 1000 Pa) at (**b**) 1830, (**c**) 1980 and (**d**) 2190 K.

Figure 3 presents surface images taken using SEM of the center of the as-received (a) and oxidized (b–d) ZBM CIP samples. After the experiment at 1830 K, the initially rough sample appears to have been covered in a smooth, glassy layer (Figure 3b). There are numerous micro-porosities on the surface oxidized at 1980 K (Figure 3c), evidencing gaseous compound release. The oxide layer formed at 2190 K (Figure 3d) is rough, with the presence of small grains of oxide phases covering the bigger grains of the substrate.

Table 1 presents the atomic composition (measured using EDS) of the surfaces of the as-received and oxidized CIP samples presented in Figure 3. The presence of carbon and aluminum is due to surface contamination from the graphite components used in SPS and subsequent alumina sandblasting to remove excess surface graphite. The low oxygen content on the surface of the as-received sample is caused by residual oxidation. This amount is logically higher after further oxidation; nevertheless, the oxygen content decreases when the oxidation temperature increases. At 1830 K, the second main element is silicon, whose content is ten times higher than that on the surface of the as-received sample. No silicon is detected on the surfaces oxidized at 1980 and 2190 K, with zirconium present at a higher content instead. While boron and molybdenum are not detected in the surfaces oxidized at 1830 and 1980 K, they are found in the surface oxidized at 2190 K.

**Figure 4:**
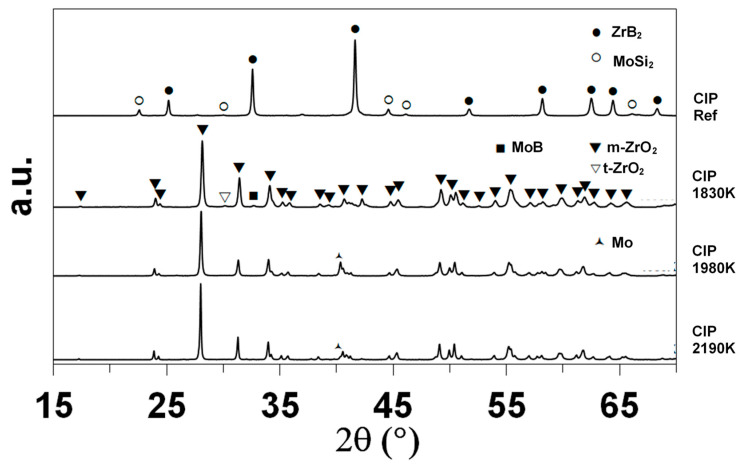
XRD diffractograms of the CIP samples, as-received and after oxidation in dissociated air at various temperatures.

XRD enabled the identification of the main crystalline compounds, as shown in Figure 4. The indexation based on the data of the International Center for Diffraction Data (ICDD) evidences the presence of ZrB_2_ (ICDD card 85-4367 or 75-0964) and MoSi_2_ (81-2167) in the as-received sample, as expected. Monoclinic zirconia (78-0048) is the main crystalline compound identified after oxidation. The sample oxidized at 1830 K contains minor amounts of tetragonal zirconia (72-2743) and molybdenum boride MoB (73-1768). The diffractograms of the samples oxidized at 1980 and 2200 K show one specific peak at 2θ = 40.51° that corresponds to elemental molybdenum.

**Figure 5:**
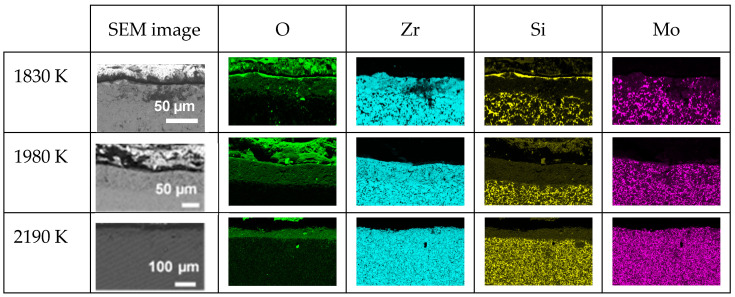
SEM images and EDS mappings of O, Zr, Si and Mo elements along the cross-sections of the three oxidized CIP samples.

Regarding the cross-section element distributions, Figure 5 presents the EDS mappings of O, Zr, Mo and Si in each oxidized CIP sample. The main observations are as follows:

- The sample oxidized at 1830 K presents a thin layer (≈3 µm) rich in silicon and oxygen on its upper surface, followed by a 37 µm thick silicon-depleted layer that also contains oxygen, with Zr, Si and Mo present and hardly any oxygen in the substrate;
- The samples oxidized at 1980 and 2190 K present a single silicon-depleted and oxygen-rich layer above the substrate. The thicknesses of this layer are 58 and 43 µm, respectively.

**Figure 6:**
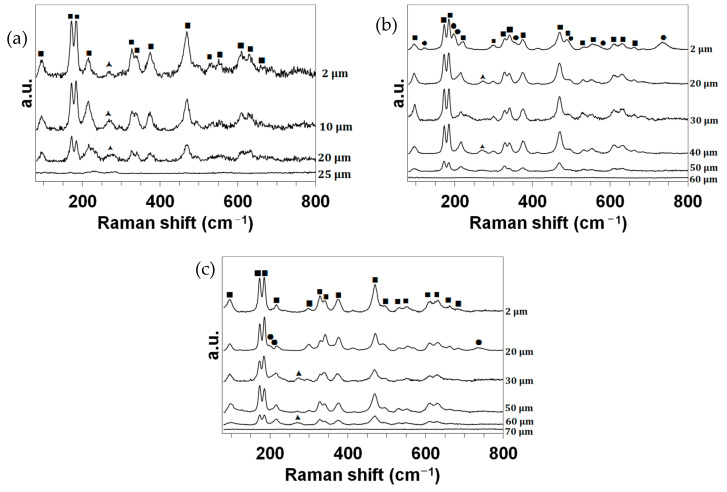
Raman spectra collected in the wavenumber range of 80 cm^−1^ to 800 cm^−1^ for the CIP samples oxidized in dissociated air at (**a**) 1830, (**b**) 1980 and (**c**) 2190 K at different depths z from the surface. ■: m-ZrO_2_. ▲: t-ZrO_2_. ●: MoO_2_.

Finally, Figure 6 presents the Raman analyses of the cross-sections. The identified peaks (precision: ±1 cm^−1^) were assigned according to the literature as follows:

- The main doublet of Raman peaks characteristic of m-ZrO_2_ compounds is well resolved and located at about 175 and 185 cm^−1^. The other Raman peaks assigned to m-ZrO_2_ compounds are observed at 99 (medium), 218 (weak), 303 (w), 330 (m), 342 (m), 377 (m), 472 (strong), 497 (w, shoulder), 533 (w), 555 (w), 612 (w), 632 (m) and 663 (w) cm^−1^ [10,11,12,13,14,15,16];
- The wide Raman band observed at about 275 cm^−1^ (w) can be assigned to the main peak of a t-ZrO_2_-like structure [13,17,18,19,20,21];
- For the CIP samples oxidized at 1980 and 2190 K, the Raman bands observed at about 125 (very weak), 201 (w), 222 (vw, shoulder), 360 (vw, sh), 567 (vw, sh) and 740 (w) cm^−1^ can be ascribed to the main peak of an m-MoO_2_ compound [22,23,24,25].

### 3.3. Characterization—RC Samples

**Figure 7:**
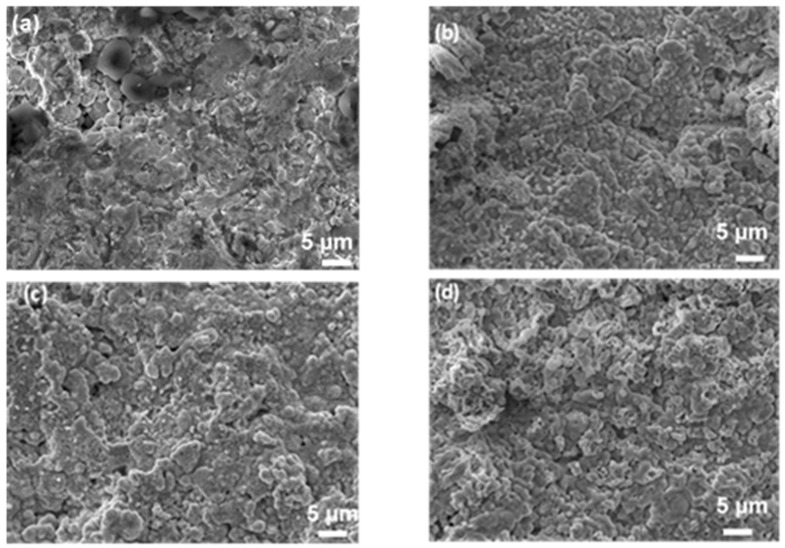
SEM images (secondary electron mode, acc. voltage: 15 kV, mag. ×2000) of the surface of RC ZBM, (**a**) as-received and after 5 min of oxidation in dissociated air (P = 1000 Pa) at (**b**) 1830, (**c**) 2000 and (**d**) 2200 K.

Figure 7 presents the surface images obtained using SEM at the center of the as-received (a) and oxidized (b–d) ZBM RC samples. The grains on the surface of the sample oxidized at 1830 K (Figure 7b) appear to be covered with a smooth oxide layer. The surfaces of the samples oxidized at higher temperatures look sharper, with the presence of cracks and porosities (Figure 7c,d).

Table 2 presents the atomic composition (measured using EDS) of the surfaces of the as-received and oxidized RC samples presented in Figure 7. Comparing Table 1 and Table 2, we observe that the reference RC sample has a higher oxygen content (due to native oxidation) and carbon content (due to contamination from the SPS and the residual carbon from the pyrolysis of the Pluronic during the de-binding process) than the reference CIP sample. Unlike the CIP samples, silicon is not detected after oxidation at 1830 K of the RC sample. Inversely, molybdenum is detected after the oxidation of the RC samples at 1830 and 2000 K but not that oxidized at 2200 K.

**Figure 8:**
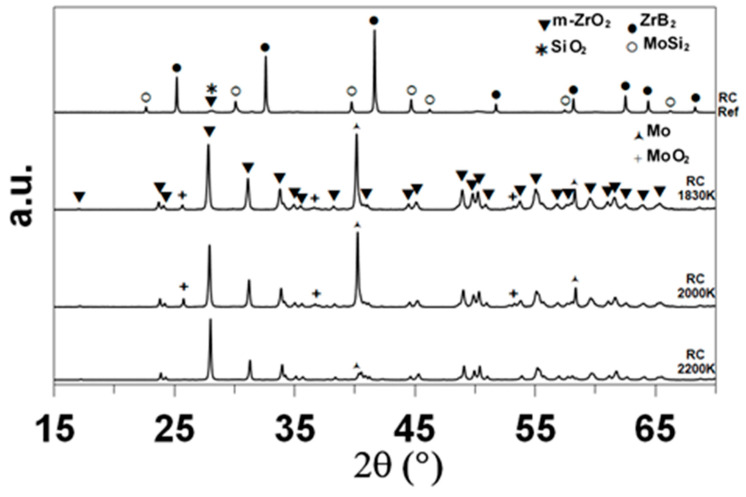
XRD diffractograms of the RC samples, as-received and after oxidation in dissociated air at various temperatures.

Figure 8 presents the XRD diffractograms of the as-received and oxidized RC samples. Besides ZrB_2_ and MoSi_2_, there are two minor phases in the as-received RC samples absent from the CIP samples, monoclinic zirconia and silica (α-quartz phase, ICDD card n°79-1913), confirming a higher degree of native oxidation in the as-received sample.

**Figure 9:**
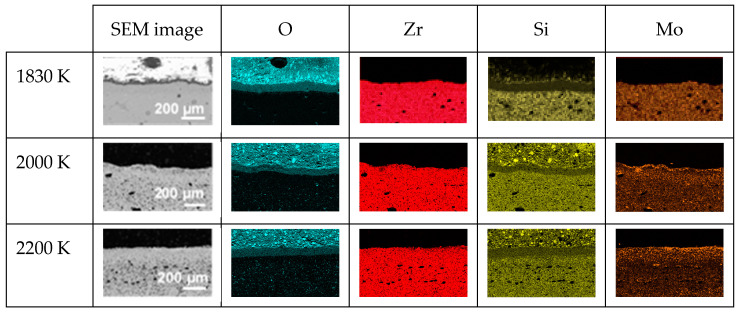
SEM images and EDS mappings of O, Zr, Si and Mo along the cross-sections of the three oxidized RC samples.

Figure 9 presents the SEM images and EDS mapping of the distribution of O, Zr, Si and Mo along the cross-sections of the three oxidized RC samples. In the three samples, we observe the presence of a silicon-depleted, oxygen-rich area whose thickness increases with temperature: 63 (1830 K), 68 (2000 K) and 75 µm (2200 K). At 2000 and 2200 K, an increased amount of molybdenum is also observed in this area.

**Figure 10:**
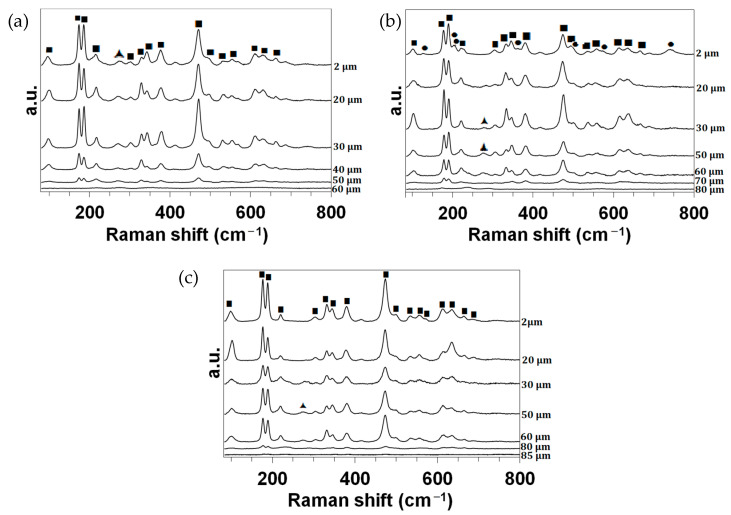
Raman spectra collected in the wavenumber range of 80 cm^−1^ to 800 cm^−1^ for the RC samples oxidized in dissociated air at (**a**) 1830, (**b**) 2000 and (**c**) 2200 K at different depths z from the surface. ■: m-ZrO_2_. ▲: t-ZrO_2_. ●: MoO_2_.

Finally, Figure 10 presents the Raman analyses of the cross-sections. The identified peaks (precision: ±1 cm^−1^) are the same as those previously assigned in Figure 6.

## 4. Discussion

All of the characterization methods we applied evidenced the formation of zirconia in the oxidized samples. Indeed, comparing experimental work and phase stability diagrams, previous authors [2,3] have reported that ZrB_2_ oxidizes above 1800 K to form solid ZrO_2_ and liquid B_2_O_3_, which volatilizes due to its high vapor pressure [26]. Equation (1) corresponds to this oxidation reaction (analogous to that for molecular oxygen [2]) with atomic oxygen:

ZrB_2_(s) + 5 O^●^ → ZrO_2_(s) + B_2_O_3_(l) (1)

According to the XRD data, primarily monoclinic zirconia is formed. Raman analyses also evidenced traces of tetragonal zirconia inside all of the oxidized samples. At 1830 K, this compound was present across the entire section of the sample (XRD also revealed the presence of this compound in the CIP sample oxidized at 1830 K). At higher temperatures, traces of this compound were localized closer to the interface with the substrate.

After oxidation in (non-dissociated) standard air at atmospheric pressure and T = 1920 and 2070 K, the ZrB_2_ + 15 vol.% MoSi_2_ samples were covered with a layer of ZrO_2_ grains encapsulated in a glassy layer of amorphous SiO_2_ [2]. The authors also observed the formation of MoB as an oxidation by-product, which can occur, according to phase stability diagrams, under reduced oxygen partial pressure [2]. Conversely, in the dissociated atmosphere, according to the surface and cross-section observations using SEM(coupled with EDS), only the CIP sample oxidized at 1830 K presented clear evidence of a 3 µm thick silica layer with some traces of MoB. The corresponding oxidation reactions (similar to those in molecular oxygen [2]) are given by Equations (2) and (3):

MoSi_2_ + 7 O^●^ → 2SiO_2_(s) + MoO_3_(g) (2)

(Zr_1−x_Mo_x_)B_2_ + (5−7x/2) O^•^ → (1−x)ZrO_2_(s) + xMoB(s) + (1−x/2)B_2_O_3_(l/g) (3)

However, the CIP samples oxidized at 1980 and 2190 K did not appear to contain any silica layers, and peaks of molybdenum appeared in the XRD results. We can suppose that at higher temperatures, MoSi_2_ partially oxidized according to Equations (4) and (5):

MoSi_2_ + 5 O^●^ → 2SiO(g) + MoO_3_(g) (4)

MoSi_2_ + 2 O^●^ → 2SiO(g) + Mo(s) (5)

The depletion of Si, associated with the continuous presence of Mo observed in the cross-sectional EDS mappings, corroborates this hypothesis, which is also supported by the fact that the variation in mass does not increase with temperature (Figure 2), while the thickness of the oxidized layer does. The transition temperature between passive (formation of SiO_2_) and active (formation of SiO) oxidation was previously determined in standard and dissociated air for sintered and CVD SiC [27]. For dissociated air at P = 1000 Pa, this temperature was 1800 K for sintered SiC, which is very close to the temperature observed for the ZrB_2_-MoSi_2_ samples in this work.

Monoclinic MoO_2_ was also identified in the superficial layers of the oxides formed at 1980 and 2190 K in the Raman spectra, although this compound was not detected at depths beyond 20 µm. This localization close to the surface supports the hypothesis that this phase is formed according to the condensation of gaseous MoO_3_ (produced by the reaction in Equation (4)) during the cooling of the samples inside the reactor.

The oxidation behavior of the RC samples differed from that of the CIP samples in the following respects:

- The oxide layer is thicker. The cross-section analyses evidence an oxide thickness of 63 to 75 µm for the RC samples, compared to 40 to 58 µm for the CIP samples. This difference can be correlated with the difference in the density of the substrate. The RC samples are less dense initially (96% vs. 99% for CIP samples) and include C in their volumetric composition as a result of the pyrolysis of the flocculant. As evidenced by the EDS data, the C evaporates (as CO_2_) during testing, contributing to additional porosity, which further facilitates the diffusion of atomic oxygen towards the interior, with it able to oxidize deeper into the RC samples;
- MoO_2_(s) also appears in the XRD diagrams for the RC samples oxidized at 1830 and 2000 K. Traces of this compound were identified in the Raman spectra at the surface of the sample oxidized at 2000 K. Again, the formation of this compound is attributed to the condensation (during the cooling phase) of gaseous MoO_3_, which forms according to Equations (2) and (4);
- The silica present in the as-received RC sample did not remain in the RC sample oxidized at 1830 K. It is possible that glassy silica formed inside the oxide layer at the grain boundaries due to the lower density of the RC samples and that these phases were too thin to clearly identify using SEM.

Regarding previous investigations reported in the scientific literature, there are very few examples of the same compounds being oxidized under similar conditions. In a recent article [28], Saha et al. investigated the cyclic oxidation of ZrB_2_ + 20 vol.% MoSi_2_ for 6 h in air at temperatures from 1373 to 1673 K and identified the presence of ZrO_2_ and SiO_2_, together with molybdenum oxide. Cyclic oxidation of the samples followed linear oxidation kinetics from 1373 to 1623 K, while at 1673 K, the oxidation kinetics were parabolic due to the protective action of SiO_2_. These authors expressed interest in continuing these investigations at higher temperatures, as undertaken here. We can confirm that silica forms at up to 1800 K but disappears at higher temperatures due to active oxidation. In future studies, we will also analyze cyclic oxidation in a dissociated atmosphere to identify whether oxidation remains protective at 1800 K and to analyze the evolution of the mechanical properties of the surface under oxidation.

## 5. Conclusions

In the course of our studies, the ZrB_2_/MoSi_2_ samples demonstrated different oxidation behaviors in dissociated air depending on the temperature and elaboration technique applied. The oxide layers constituted zirconia, as well as silica at temperatures around 1800 K. Above 1800 K, silica was no longer present. Traces of molybdenum oxide were also identified close to the surface of the oxidized samples, resulting from the condensation of gaseous molybdenum trioxide during cooling. The elaboration technique affects the density of the material, and therefore, a less dense material permits deeper oxidation.

As silica is expected to protect ceramics from deeper oxidation, the maximal operating temperature for a thermal shield containing ZrB_2_ + 20 vol.% MoSi_2_ would be 1800 K, with the growth of a protective silica layer favoring diffusion-controlled oxidation with semi-parabolic kinetics [29]. We will apply thermal cycling in future experimental campaigns in order to validate this hypothesis further. We will then incorporate micro-channels into ceramic samples as part of an active cooling system to ensure that we can maintain the temperature below 1800 K under service conditions (e.g., during re-entry) and reduce oxidation damage to the ceramic. This will enable the fabrication of non-ablative thermal protection systems for reusable spacecraft vehicles that will greatly facilitate their reusability and drastically reduce the maintenance costs and time between uses. Moreover, non-ablative thermal shields are a more environmentally friendly solution, especially when fabricated by applying consolidation techniques that are fast and less energy-intensive, like the pressureless SPS used in this study.

## Figures and Tables

**Table 1:** Atomic composition of the surfaces of the CIP samples presented in Figure 3.

	O	Zr	Mo	B	Si	C	Al
As-received	13.0	12.4	1.9	45.4	2.1	23.9	1.3
1830 K	70.3	3.5	/	/	21.0	4.5	0.7
1980 K	55.9	27.9	/	/	/	16.2	/
2190 K	28.7	15.5	2.9	29.2	/	23.1	0.6

**Table 2:** Atomic composition of the surfaces of the RC samples presented in Figure 6.

	O	Zr	Mo	B	Si	C	Al
As-received	19.9	10.2	2.5	28.2	1.5	36.4	1.4
1830 K	53.4	16.5	8.4	/	/	21.7	/
2000 K	54.4	14.9	13.8	/	/	17.0	/
2200 K	47.0	23.2	/	13.4	/	16.4	/

## Data Availability

The research data presented here are available from the corresponding author on request.
